# Recovery of rare earth elements by nanometric CeO_2_ embedded into electrospun PVA nanofibres†

**DOI:** 10.1039/d1ra02097h

**Published:** 2021-05-28

**Authors:** Daniele Comandella, Walter Bonani, Jorge Bañuls Ciscar, Jessica Ponti, Marco Cologna, Karin Popa, Douglas Gilliland

**Affiliations:** European Commission, Joint Research Centre (JRC) Ispra Italy douglas.gilliland@ec.europa.eu; European Commission, Joint Research Centre (JRC) Karlsruhe Germany

## Abstract

Rare earth elements (REEs) are critical raw materials with a wide range of industrial applications. As a result, the recovery of REEs *via* adsorption from REE-rich matrices, such as water streams from processed electric and electronic waste, has gained increased attention for its simplicity, cost-effectiveness and high efficacy. In this work, the potential of nanometric cerium oxide-based materials as adsorbents for selected REEs is investigated. Ultra-small cerium oxide nanoparticles (CNPs, mean size diameter ≈ 3 nm) were produced *via* a precipitation-hydrothermal procedure and incorporated into woven–non-woven polyvinyl alcohol (PVA) nanofibres (*d* ≈ 280 nm) *via* electrospinning, to a final loading of ≈34 wt%. CNPs, CNP–PVA and the benchmark material CeO_2_ NM-212 (JRCNM02102, mean size diameter ≈ 28 nm) were tested as adsorbents for aqueous solutions of the REEs Eu^3+^, Gd^3+^ and Yb^3+^ at pH 5.8. Equilibrium adsorption data were interpreted by means of Langmuir and Freundlich data models. The maximum adsorption capacities ranged between 16 and 322 mg_REE_ g_CeO2_^−1^, with the larger value found for the adsorption of Yb^3+^ by CNP. The trend of maximum adsorption capacity was CNPs > NM-212 > CNP–PVA, which was ascribed to different agglomeration and surface area available for adsorption. Langmuir equilibrium constants *K*_L_ were substantially larger for CNP–PVA, suggesting a potential higher affinity of REEs for CNPs due to a synergistic effect of PVA on adsorption. CNP–PVA were effectively used in repeated adsorption cycles under static and dynamic configurations and retained the vast majority of adsorptive material (>98% of CeO_2_ retained after 10 adsorption cycles). The small loss was attributed to partial solubilisation of fibre components with change in membrane morphology. The findings of this study pave the way for the application of CNP–PVA nanocomposites in the recovery of strategically important REEs from electrical and electronic waste.

## Abstract 2

Electrospun poly(vinyl alcohol) membranes with nano CeO_2_ could effectively recover rare earth ions from model water solutions.

## Graphical abstract

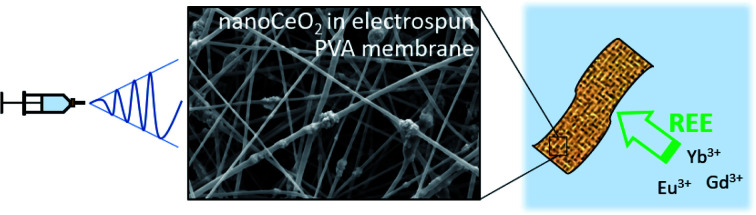

## 1. Introduction

Rare earth elements (REEs) are elements with unique physicochemical properties that are indispensable for the production of technological products such as magnets, lamp phosphors and metal alloys in catalysts and rechargeable batteries.^1–3^ The demand of REEs has been steadily increasing in the past decades,^2,4^ driven by the growing need of hi-tech goods and the implementation of sustainable manufacturing policies. Also, because of political tensions and monopolistic supply conditions,^5^ since 2010 the European Commission has included REEs in the list of critical raw materials, *i.e.* materials with high economic importance and high supply risk.^6^ A substantial improvement in the recycling rates of REEs from waste electrical and electronic equipment (or WEEE) has been therefore considered a strategic necessity^1^ to secure REEs supply, minimize waste generation and protect the environment.^7^ Nonetheless, recuperation or recycling is not yet a substantial part of REEs' supply chain,^8^ with recycling primarily directed on pre-consumer scrap/residues^9,10^ or large post-consumer parts with high REEs content (such as permanent magnets). The recuperation of REEs from post-consumer end-of-life technological products in waste electrical and electronic equipment represented only 1% of total recycled REEs in 2011, due to a combination of inefficient collection, technological difficulties and, especially, a lack of incentives.^11,12^ Despite recent advances in laboratory-scale tests^13,14^ such processes are not yet implemented at industrial level. Therefore, technologies that can efficiently recover REEs from WEEE such as printed circuit boards,^15^ hard disk drives^16,17^ or lamp phosphors^12,18^ have gained increased attention as alternative source of REEs.^19^ Owing to the physicochemical similarities between lanthanides and actinides, improved processes for the recovery of REEs could have important applications in other fields. For example, REEs such as europium and neodymium have been used as non-radioactive, non-hazardous homologues of trivalent actinide elements in the detoxification of radioactive wastewaters.^20–22^

A well-established process for the extraction of REEs from WEEE is the so-called hydrometallurgical route, where metals are leached out from the solid matrix into an aqueous solution *via* a combination of acidic and oxidative treatments.^7,18,23,24^ The final step of the process features the separation, purification, and concentration of REEs by using solvent extraction,^25,26^ chemical precipitation,^27,28^ ion exchange,^29^ electrochemical methods^30^ or biosorption.^31^ Among these methods, adsorption emerged as a simple, cost-effective, non-toxic, waste-free approach to separate and concentrate REEs.^32^ A variety of adsorptive materials have been used, such as polymers,^51^ clays,^33^ MOFs,^34^ carbon-based materials,^35,36^ natural-derived fibres^37,38^ and metal oxide particles.^22,39,40^ Nanosized cerium oxide (nanoceria) with a fluorite-like structure is a versatile material with various technological applications^41^ that has recently proved to be an effective adsorbent for metal ions.^42–46^ The high surface area of CeO_2_ nanoparticles combined with its variable morphologies is believed to enhance the removal of metal ions from water.^42,44,45,47^ Moreover, the quite unique ability of CeO_2_ to easily cycle between +3 and +4 oxidation states together with the generation of oxygen vacancies is an appealing property that could be used during the adsorption process to enhance the efficacy and selectivity of adsorbents. For example, cerium oxides nanocrystals were found to effectively capture U(vi) from aqueous streams,^47–49^ and this was attributed to the mixed surface valence, varying morphology and geometry of CeO_2_ nanoparticles.^47^

However, despite the known physicochemical similarity between actinides such as U(vi) and lanthanides, which would suggest a similar uptake from CeO_2_, very few studies on the adsorption of REEs by CeO_2_ nanoparticles were carried out so far.^50^ Moreover, the above-reported adsorption studies have mostly used water-suspended nanoparticles, a configuration that might prove to be ineffective in real-world application. Suspended nanoparticles are intrinsically prone to agglomeration and this can result in a decrease of their adsorption performance and an inefficient separation from the medium due to sedimentation. In batch processes, where adsorption takes place into a closed vessel, additional costs could arise from final solid–liquid separation by filtration or centrifugation. In dynamic processes, where a solution of REE ions flows into a system containing the adsorbent (*e.g.*, through a packed bed), very small nanoparticles could be lost by passing through ordinary filters or generate overpressure by blocking the system's outlet.

These problems can be solved by incorporating metal oxide nanoparticles into solid polymer membranes. Nanocomposites made of porous membranes and finely disperse inorganic nanoparticles are considered to have potential in the separation or pre-concentration of REEs from streams.^51^ Ideally, the membrane should not only confine and stabilises the adsorbent, but also have a synergistic effect on adsorption. Electrospinning has emerged as a versatile and simple technique to produce micro-porous membranes made of nanofibres networks with large surface-to-volume ratio and high porosity by using a wide range of polymer materials.^52,53^ Owing to their micro- or nano-porous structure and their high mechanical strength, electrospun fibrous membranes represent a suitable material for microfiltration applications. Because of that, they have been used in the removal or in the pre-concentration of heavy metal ions as such^54,55^ and in combination with inorganic nanoparticles.^56–59^

This study investigates the potential of CeO_2_ nanoparticles as adsorbent material for the recovery of REE ions from aqueous solutions. To this end, 3 nm CeO_2_ nanoparticles (CNPs) were produced and incorporated in poly(vinyl alcohol) (PVA) nanofibrous membranes *via* electrospinning, thereby originating CNP–PVA nanocomposites. PVA was selected for its frequent use in the production of electrospun nanocomposites, good mechanical properties, stability over a wide pH range and the ability to swell in water,^60^ which would facilitate the diffusion of REE ions towards the surface of CeO_2_ nanoparticles. Unsupported CNPs and CNP–PVA were tested in the static and dynamic adsorption of selected REE ions (Eu^3+^, Gd^3+^, Yb^3+^) from aqueous solutions. Their adsorptive properties were compared with the benchmark material CeO_2_ NM-212, a representative test material for manufactured nanoceria.^61^ Equilibrium adsorption data were interpreted *via* Langmuir and Freundlich data models in order to determine the materials' adsorption properties such as the maximum adsorption capacity.

## 2. Materials and methods

Cerium(iv) ammonium nitrate (NH_4_)_2_Ce(NO_3_)_6_, ammonia solution 25% in water, poly(vinyl alcohol) powder (PVA – Mowiol® 56–98, >98 mol% hydrolysis, *M*_W_ = 195 kDa), poly(acrylic acid) sodium salt (PAA, *M*_W_ = 5 kDa), Triton *x*-100 and Eu, Gd, Yb nitrates were purchased from Merck (Darmstadt, Germany). The material CeO_2_ NM-212 (henceforth: NM-212) was provided by the JRC Nanomaterials Repository of the European Commission at the Joint Research Centre (JRC, Ispra, Italy).

### 2.1 Production of materials

Cerium oxide nanoparticles (henceforth, CNPs) were produced *via* a precipitation-hydrothermal route as already reported elsewhere.^62,63^ Briefly, a 0.1 M cerium(iv) ammonium nitrate solution was prepared by dissolving the salt in deionized water. Then, an aliquot of ammonia solution was added to the solution under constant stirring to promote the precipitation of hydrous CeO_2_. The ammonia to cerium molar ratio (here = 4) is crucial as the excess of hydroxide ions controls the nanoparticle's size. After 2 h, the solid was recovered *via* centrifugation and washed with deionized water and ethanol. Then, the solid was re-suspended in deionized water and heated up (0.5 °C min^−1^ to 120 °C, holding time = 30 min) using a microwave digestion system (Discover SP, CEM corporation, 200 W). Finally, CNP powder was recovered *via* centrifugation, washed with deionized water and ethanol, and stored either as powder or as suspension in deionized water.

Electrospun nanofibrous membranes with incorporated CNPs (henceforth: CNP–PVA) were produced with an electrospinning apparatus (Linari Engineering, Pisa, Italy) operating at 20 kV. In a standard procedure, a CNPs suspension (*c*_CNP_ = 25 wt%, *c*_PAA_ = 0.5 wt%) was mixed with a 11 wt% PVA solution (volume to volume ratio: 1 to 3). PAA was added in order to stabilise the suspension and prevent sedimentation.^63^ A small amount of the non-ionic surfactant Triton x-100 was added to a final concentration < 0.1 wt% in order to facilitate the electrospinning deposition by decreasing the suspension's surface energy. Then, the suspension was delivered to a stainless steel 22 G blunt tip needle (Hamilton, Bonaduz, Switzerland) by a motorized syringe (Harvard Apparatus, Holliston, MA, USA) operating at 1 mL h^−1^. Deposition occurred onto a grounded aluminium collector with cylindrical shape (radius = 4 cm), rotating at 60 rpm, resulting into the production of membranes of various thicknesses (from 50 to 100 μm) consisting of woven–non-woven randomly oriented nanofibres. Electrospun nanofibrous membranes without filler (henceforth, PVA) were produced with the same procedure at a different flow (1.2 mL h^−1^) and voltage (24 kV).

### 2.2. Characterisation

#### 2.2.1. Characterisation of CNPs

##### Crystallinity and particle size

Crystalline phase of CNPs was determined *via* powder X-ray diffraction (XRD) with a Bruker D8 Discover diffractometer operated with a monochromatic Cu K alpha radiation from a W source (1.5406 Å, 40 kV, 40 mA), using a step scan mode in the range from 20 to 90 degrees, with a step of 0.02° and 4 s per step. The mean crystallite sizes were determined from the diffraction peaks (111), (200), (220), (311) by applying the Scherrer equation:1
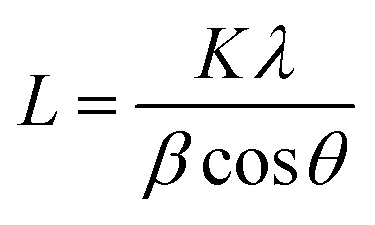
where *L* is the size of coherently diffracting domains, *K* is the Scherrer shape constant (0.9 for spherical structures), *λ* is the incident X-ray wavelength (Cu K_α_ = 0.15406 nm), *θ* is the Bragg angle, and *β* is the peak full width at half maximum intensity, determined by fitting the peaks with pseudo-Voigt line shapes. Because CeO_2_ is a semiconductor, an “optical” particle size can be derived by determining its optical band gap from its UV-visible absorption spectrum.^64^ UV-vis spectra were acquired with a Thermo Scientific Evolution 350 UV-visible spectrophotometer equipped with glass cuvettes (1 cm path length) at room temperature in the wavelength *λ* range 270–700 nm. The optical band gap *E*_g_ was found from the CNPs absorption coefficient *α* by plotting the so-called Tauc plot and determining the intersection of the extrapolated linear portions with the *x*-axis.^65^ Finally, the particle size was be determined with eqn (2)2
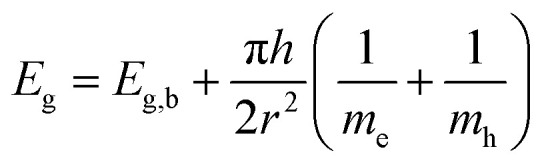
where *E*_g,b_ is the bulk band gap for ceria (3.19 eV), *r* is the particle radius (m), *h* is the reduced Plank constant, and *m*_e_ and *m*_h_ are the effective masses of electron and hole, respectively. Please refer to Section S1† for the detailed calculation process. The CNPs particle size distribution, expressed as the minimum Feret diameter, was determined by Transmission Electron Microscopy (TEM) with a JEM 2100 (JEOL, Milan, Italy). TEM samples were prepared by placing a droplet of a CNP suspension in deionized water onto a formvar/carbon – coated 200 mesh copper grid (Agar Scientific, USA), which was previously hydrophilized by a short glow discharge treatment (30 s, 10 mA) with a Leica EM ACE200 (Leica Microsystems, Milan, Italy). After drying, TEM micrographs were obtained at 120 kV with a magnification of 4000 to 40 000×. Finally, images were analysed by ImageJ software (http://rsb.info.nih.gov/ij/) using the Nanodefine ParticleSizer plugin (https://imagej.net/ParticleSizer) to determine the particles' minimum Feret diameter.

##### Specific surface area SSA

Brunauer–Emmett–Teller (BET) specific surface area (*S*_BET_) was measured by nitrogen adsorption at 77 K (Gemini VII, Micromeritics, US) by fitting at least five points in the linear isotherm range. The samples were degassed prior analysis in order to remove adsorbed water and other impurities at 180 °C for 3 h under a 50 mTorr vacuum.

##### Agglomeration and surface charge

The size of agglomerates of water-suspended CNPs was determined *via* asymmetric field flow fractionation (AF-FFF) with an AF2000 system (Postnova Analytics, Landsberg am Lech, Germany). The system was equipped with UV-visible (PN3211, Postnova Analytics, *λ* = 300 nm) and dynamic light scattering DLS (Zetasizer Nano ZS, Malvern Instruments, UK) detectors. The fractionation channel was equipped with a 350 μm spacer and a 10 kDa cut-off regenerated cellulose membrane. Eluent was a 0.02 wt% solution of Novachem™ at pH 9.5. The focus step was conducted with injection flow = 0.25 mL min^−1^, injection time = 4 min, cross flow rate = 0.8 mL min^−1^. The elution step was conducted with a cross flow rate from 0.8 to 0.05 mL min^−1^ (linear decrease) for 50 min. Prior to injection, CNP suspensions (*c*_CeO_2__ = 250 mg L^−1^) were sonicated for 10 min at 24 kHz (VialTweeter, Hielscher Ultrasonics GmbH, Germany) and then injected (20 μL) into the AF-FFF channel. The surface charge of CNP suspensions (*c*_CeO_2__ = 0.5 mg L^−1^) was investigated by determining its zeta potential (Zetasizer Nano-ZS, Malvern Instruments, UK).

#### 2.2.2. Characterisation of CNP–PVA

##### Morphology

The morphology of electrospun fibres was studied by transmission electron microscopy TEM (JEM 2100, JEOL, Italy) and scanning electron microscopy SEM (VersaTM 3D DualBeamTM, FEI, US). TEM samples were prepared by electrospinning, placing a TEM grid between the delivering needle and the collector, resulting in the deposition of a number of fibres onto the grid. For SEM measurements, electrospun membranes were sputtered with gold and SEM images were collected with an acceleration voltage of 5 keV. Micrographs were analysed with ImageJ software to determine nanofiber size and distribution from a minimum of 150 fibres per sample. The presence and distribution of CNPs in the PVA membranes were investigated by Time-of-Flight Secondary Ion Mass Spectrometry (ToF-SIMS), performed using a ToF-SIMS IV system (IONTOF GmbH, Muenster, Germany). ToF-SIMS is a powerful surface technique that can provide chemical information about the morphology of the upper and inner layers of a polymer membrane. Owing to its high sensitivity, high spatial resolution and mass resolution, ToF-SIMS can offer label-free chemical imaging capabilities down to nanometre scale.^66–68^ Mass spectra were acquired using a 25 keV Bi_3_^+^ primary ion beam operated with the instrument optimised for maximum mass resolution (*m*/Δ*m* ∼ 3000–4000 in high-current bunched mode, pulse width = 1 ns, beam diameter ≈ 3 μm) with a target current of 0.7 pA. Secondary ion images were acquired with the instrument optimised for maximum lateral resolution (*m*/Δ*m* < 500 in fast imaging mode, pulse width = 100 ns, beam diameter ≈ 0.4 μm) with a target current of 0.05 pA. The primary ion dose was kept below the so-called static limit (10^13^ ions per cm^2^) minimising surface damage. An analysis area of 100 × 100 μm^2^ was used for imaging, and image resolution was set to 512 × 512 μm^2^ leading to an approximate pixel size of 200 × 200 nm^2^. Surface mass spectra in the mass resolution mode were taken in order to identify ion species present in the specimen surface. Mass spectra were calibrated using the same peak list and peak assignments were performed based on the measured ion mass compared to the calculated one. This facilitated the interpretation of mass spectra obtained using the fast-imaging mode where mass resolution is significantly low. Spectra and images were processed using the SurfaceLab software V6 (IONTOF GmbH).

##### Composition and stability

The content of nanoceria in CNP–PVA was measured by thermally digesting small pieces of membrane (≈5 mg) in a 3 v/v% H_2_O_2_ solution under UV light (60 min at 80 °C) and by determining the cerium content in the resulting suspensions *via* total reflection X-ray fluorescence TXRF (S4 T-STAR, Bruker, US). The thermal stability of CNP–PVA nanocomposites was investigated *via* thermogravimetric analysis (TGA) with a STA 449 C Jupiter (Netzsch, Germany) by heating up the sample from 40 to 700 °C at 10 °C min^−1^. Further insights in the CNP–PVA composition were obtained from attenuated total reflectance Fourier-transform infrared spectroscopy (ATR-FTIR) with an Alpha FT-IR spectrometer (Bruker, Germany) equipped with ZnSe crystal. FT-IR spectra were acquired at room temperature in the wavenumber range from 600 to 4000 cm^−1^ with a resolution of 4 cm^−1^. To investigate the behaviour of electrospun membranes in water, small pieces of membranes (≈10 mg) were immersed in deionized water at room temperature. After four hours, the membranes were removed, dried (15 min at 80 °C) and weighed again to determine a potential weight loss. The thermal stability and surface composition of water-exposed membranes were then evaluated from TGA and ATR-FTIR measurements.

### 2.3. Adsorption tests

#### 2.3.1. Static adsorption tests

Unless otherwise stated, adsorption experiments performed in this work were conducted in deionized water at pH 5.8 at room temperature (18 °C). For static tests, a suitable amount of adsorbent (CNPs, NM-212, or CNP–PVA) was added to 3 mL adsorbate solution (*c*_REE_ = 0.5 to 150 mg L^−1^; REEs: Eu^3+^, Gd^3+^, Yb^3+^) to a final nanoceria concentration *c*_CeO2_ ≈ 0.25 g L^−1^. At least three replicates were prepared for each REE concentration. Then, the systems were left under agitation overnight to establish the adsorption equilibrium. After that, the water phase was analysed by TXRF to determine the REEs concentration at equilibrium. In the case of water-suspended nanoceria (CNPs and NM-212), the water phase was separated from nanoceria by centrifugation with ultra-centrifugal filters (Amicon 30 kDa filters, Millipore, US) prior to TXRF analysis. The equilibrium adsorption capacity *q*_e_ (mg_REE_ g_CeO2_^−1^) and the fraction of adsorbed REEs (REE %) were then calculated from eqn (3) and (4), respectively:3
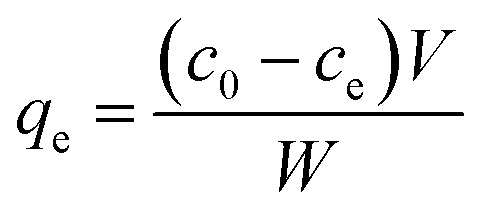
4
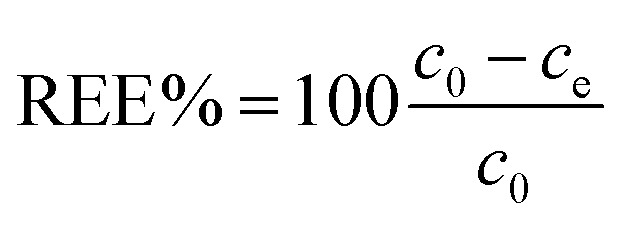
where *V* is the system volume (L), *W* is the adsorbent mass (g), and *c*_0_ and *c*_e_ (mg L^−1^) are the REE concentrations determined at time 0 and at equilibrium, respectively. To investigate the effect of pH on adsorption, the pH of the system was adjusted with 0.1 M HNO_3_ or NaOH solutions. To obtain the adsorption isotherms for every adsorbent, *q*_e_ and *c*_e_ were determined with various initial REE concentrations (from 2 to 150 mg L^−1^). Then, the two-parameter Langmuir and Freundlich models (eqn (5) and (7), respectively) were used to correlate experimental adsorption data. The Langmuir model is routinely used to describe the adsorption process and assumes a monolayer adsorption on a homogeneous surface. The Langmuir model is expressed by eqn (5).5
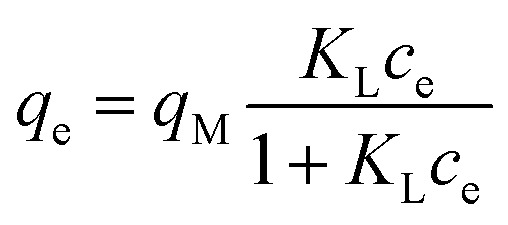
where *q*_M_ is the equilibrium and maximum adsorption capacity of sorbate onto sorbent (mg_REE_ g_CeO2_^−1^), *c*_e_ is the residual equilibrium concentration of the adsorbate in the bulk water phase, and *K*_L_ (L mg_REE_^−1^) is the Langmuir adsorption equilibrium constant. The so-called separation factor *R*_L_ was determined based on *K*_L_ and initial REE concentration *c*_REE,*t*=0_ as6
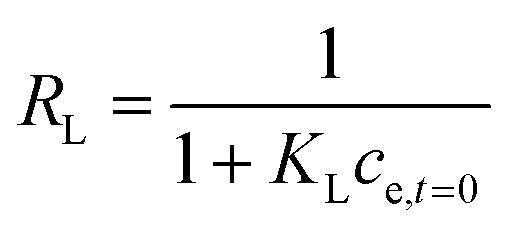


In the Freundlich model, the adsorption of adsorbate occurs on a heterogeneous surface by multilayer sorption. The non-linear Freundlich equation is7
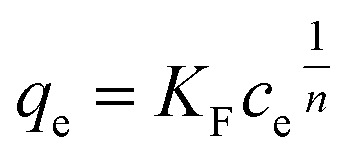
where *K*_F_ (mg_REE_ g_CeO_2__^−1^) (mg_REE_ L^−1^)^*n*^ is the Freundlich adsorption equilibrium constant and *n* is a correction factor or sorption intensity. Values of *q*_M_, *K*_L_, *K*_F_ and *n* were determined by non-linear fitting with the data analysis software Origin (v. 9.1, OriginLab, US). The Freundlich model is empirical and does not hold at high fractional coverage of the adsorbent's surface. Therefore, only *q*_e_ values that were <80% of maximum of adsorption capacity *q*_M_ (determined experimentally or *via* the Langmuir fitting) were included in the fit.

#### 2.3.2. Repeated use of adsorbents

##### Static arrangement

The adsorbent (either CNP–PVA or an aliquot of CNP suspension) was added to 3 mL of Eu^3+^ solution (5 mg L^−1^) to a final *c*_CeO_2__ = 0.25 g L^−1^ (for CNPs) or 0.5 g L^−1^ (for CNP–PVA). After equilibration, the water phase was separated from the adsorbent and analysed for its europium and cerium content. Then, the adsorbent was once again added to fresh Eu^3+^ solution and the process repeated for 10 adsorption cycles. For each step *n*, of the adsorbed capacity *q*_e,*n*_ and the amount of ceria lost from the adsorbent were determined. Then, the cumulative adsorption capacity *q*_c,*n*_ was calculated as:8
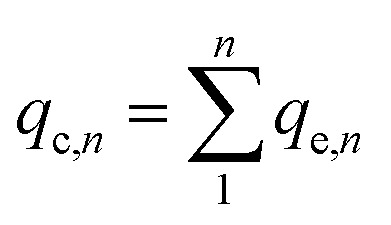


The trend of *q*_c,*n*_ and the loss of CeO_2_ were used to evaluate the suitability of adsorbents under repeated use. At the end of the experiment, Eu^3+^-loaded adsorbents were contacted with 0.01 M HNO_3_ to promote desorption of Eu^3+^. After equilibrating overnight, the amount of desorbed Eu^3+^ was determined *via* analysis of the water phase with TXRF.

##### Dynamic arrangement

A custom-made laboratory-scaled flow-through system was prepared. A CNP–PVA membrane (*m* ≈ 5 to 20 mg) was placed between two PTFE frits and housed into a syringe. Then, a Eu^3+^ solution (*c*_0_ = 3, 20 or 40 mg L^−1^) was pumped through the as-made filtering unit with a syringe pump (flow = 0.1 mL min^−1^). Please see Section S2† for a sketch of the system. The outlet flow was sampled at given time points and analysed *via* TXRF to determine the Eu^3+^ concentration in solution (*c*_*t*_). As the concentration in the system feed *c*_0_ was kept constant throughout the experiment, the system can be approximated to a continuous flow reactor and the adsorption rate *r* (μmol g^−1^ h^−1^) can be found by adapting the performance equation of plug-flow reactors^69^ as:9
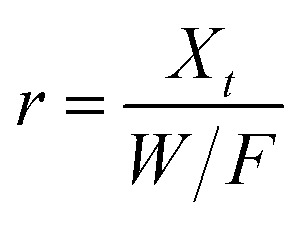
where *W* is the adsorbent mass (g), *F* is the Eu^3+^ molar flow (μmol h^−1^), and *X*_*t*_ is Eu^3+^ adsorbed conversion at time *t* (*X*_*t*_ = 1 − *c*_*t*_/*c*_0_). Eqn (9) shows that the adsorption rate could be monitored over time by determining the Eu^3+^ concentration in the outlet flow.

## 3. Results and discussion

### 3.1. Characterisation

A summary of the characterisation of CNPs and CNP–PVA is given in Table 1. Please refer to the ESI Section S3† for the characterisation of the benchmark material NM-212.

**Table 1:** List of physicochemical properties of CNPs and CNP–PVA

Physicochemical property	Technique	Measured property	Measured value
**Cerium oxide nanoparticles CNPs**			
Particle size	XRD	*d*_XRD_ average size of the crystalline domain	2.6 ± 0.2 nm
	TEM	*d*_TEM_ median value of the particle size distribution	3.0 ± 1.0 nm
	UV-vis	*d*_UV_ optical band gap size	≈2.3 nm
Size of agglomerates	AF-FFF/DLS	*d*_H_ *z*-average hydrodynamic diameter	163.9 ± 30.3 nm
SSA	N_2_ adsorption	*S*_BET_ surface area determined *via* BET model	203.7 ± 1.6 m^2^ g^−1^

**Electrospun membranes CNP–PVA**			
Size of agglomerates	TEM	*d*_TEM_ average diameter	from 50 to 2400 nm
Nanofiber size	SEM	*d*_SEM_ average diameter of nanofibres	280 ± 80 nm
Composition	TGA	Concentration by weight	CNPs: 37%, PVA: 63%
	Digestion + TXRF	Concentration by weight	CNPs: 33.6 ± 6.8%

#### 3.1.1. Characterisation of CNP

##### Crystallinity and particle size

**Fig. 1:**
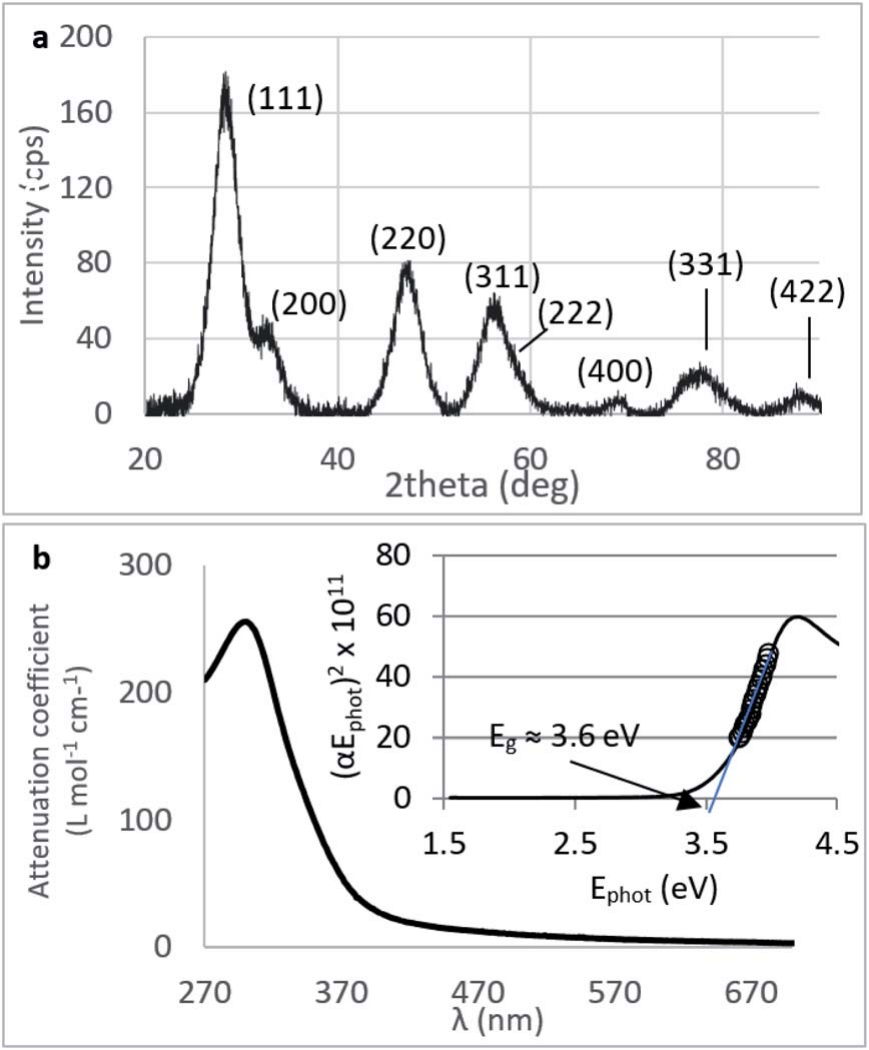
(a) XRD diffraction pattern of CNPs (background subtracted) displaying the miller indices associated with each peak. (b) UV-vis spectrum of CNPs and corresponding Tauc plot (*c*_CeO_2__ = 30 mg L^−1^).

**Fig. 2:**
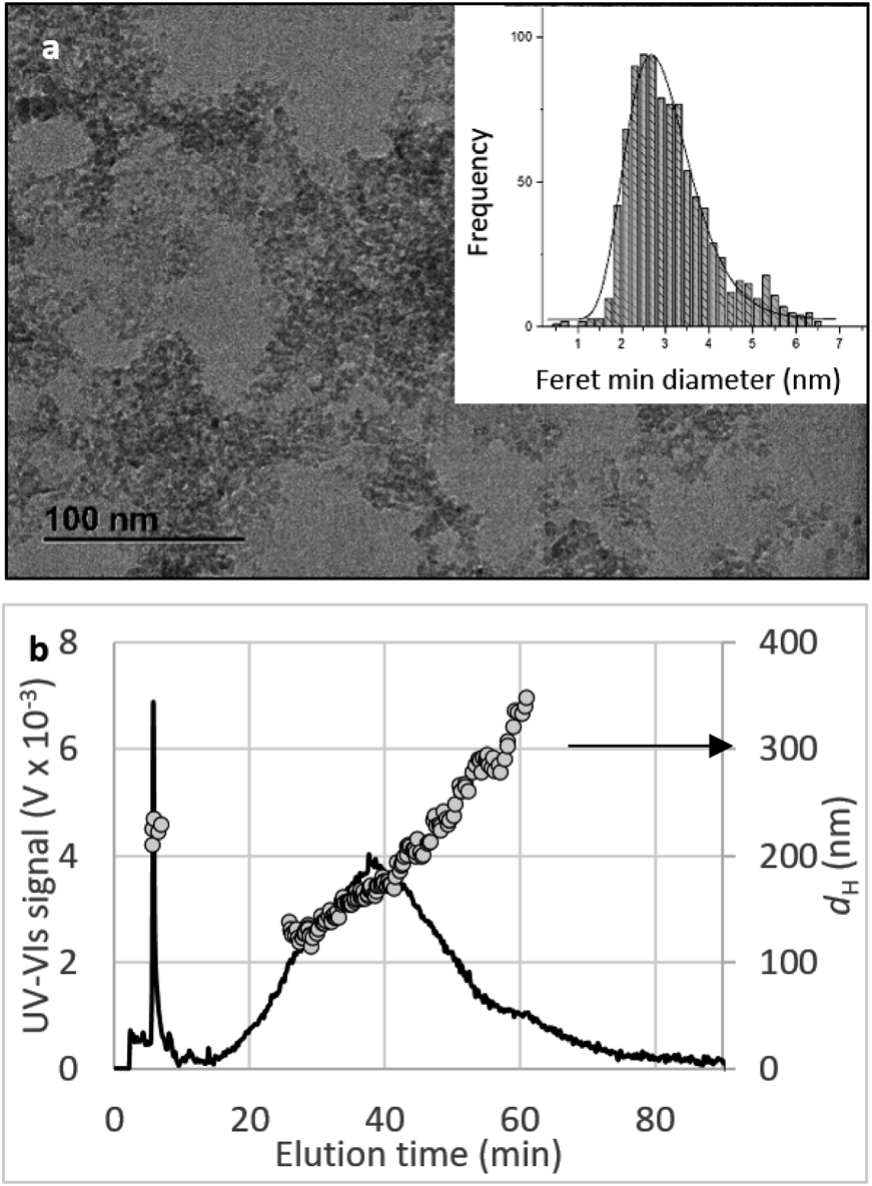
Size characterisation of CNPs. (a) TEM image of CNPs and the corresponding particle size distribution (particles counted: 942). (b) UV-vis elugram (continuous line) and *d*_H_ calculated by DLS (grey dots) from AF-FFF measurements. Injected volume: 20 μL, pH = 9.5, *c*_CeO_2__ = 0.25 g L^−1^.

The X-ray diffraction pattern of CNPs (Fig. 1a) is consistent with the International Centre for Diffraction Data (ICDD) database^70^ file for cerium oxide with fluorite-like crystal structure (PDF 00-001-0803). The mean crystallite size was determined from the first four diffraction peaks using eqn (1) and resulted in *d*_XRD_ ≈ 2.6 nm. The UV-vis spectrum of CNPs (Fig. 1b) exhibits a strong absorption band below *λ* = 400 nm, attributed to O_2p_ → Ce_4f_ charge transfer electronic transition,^71^ with a maximum of absorbance at *λ* ≈ 300 nm. The optical band gap found *via* the corresponding Tauc plots (Fig. 1a and S1†) was *E*_g_ ≈ 3.6 eV, which corresponded to an optical size *d*_UV_ = 2.3 nm *via*eqn (2). Finally, the particle size distribution of CNPs was determined from TEM micrographs. Fig. 2a shows an example of such micrographs, together with the corresponding particle size distribution based on the nanoparticles' minimum Feret diameter. TEM analysis showed a population of primary particles smaller than 7 nm, with a median value *d*_TEM_ ≈ 3.0 nm and a polydispersity index = 0.3. Please refer to Section S4† for additional information.

##### SSA

SSA of CNPs determined by N_2_ adsorption measurements *via* the BET plot resulted in *S*_BET_ ≈ 204 m^2^ g^−1^. This value is comparable than the one determined from the particle size distribution obtained from TEM images (≈214 m^2^ g^−1^, see Section S4†).

##### Agglomeration of nanoceria

The UV-vis/DLS elugram of water-suspended CNPs from AF-FFF measurements (Fig. 2b) shows the presence of large agglomerates with a *d*_H_ in the size range from 100 to 350 nm and an average *d*_H_ ≈160 nm. Similar results were found for NM-212 in this study (Section S3†) and in previous works with static DLS.^72^ The presence of large agglomerates was ascribed to an ineffective re-suspension of nanoceria powder into water during the preparation of CNPs (paragraph 2.1) and to unfavourable pH of the system. Stabilisation of dispersion by electrostatic repulsion is usually achieved when their zeta-potential is >30 mV, which is not the case for the suspensions used in this work. In fact, the suspension's pH (5.8) is near to its point-of zero charge pH_PZC_ (Fig. 6b).

#### 3.1.2. Characterisation of CNP–PVA

##### Morphology

**Fig. 3:**
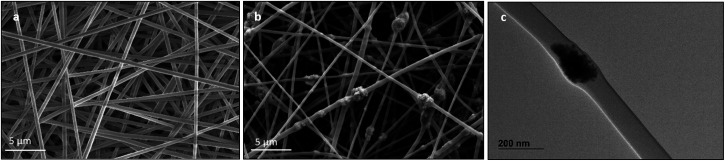
SEM image of pristine (a) PVA and (b) CNP–PVA membranes; (c) TEM image of a CNP–PVA nanofibre.

SEM and TEM images (Fig. 3) show that both PVA and CNP–PVA were highly porous membranes composed of sub-micron fibres with smooth surface and uniform individual fibre size. The average diameter of CNP–PVA nanofibres was 280 ± 80 nm, significantly smaller than for the filler-free membrane (390 ± 53 nm). The reduction of fibre size upon nanoparticle incorporation has been frequently reported and is believed to be caused by the difference in viscosity and conductivity between the PVA solution and PVA–CNP suspension before electrospinning deposition.^73^ CNP–PVA SEM micrographs (Fig. 3b) shows the presence of large, spindle-like structures with size ranging from 50 to 2400 nm. The strong contrast between the inner part of such objects and the adjacent polymer nanofibre, as observed in TEM micrographs (Fig. 3c and Section S4†), suggests that they contain the more electron-dense CNPs.

**Fig. 4:**
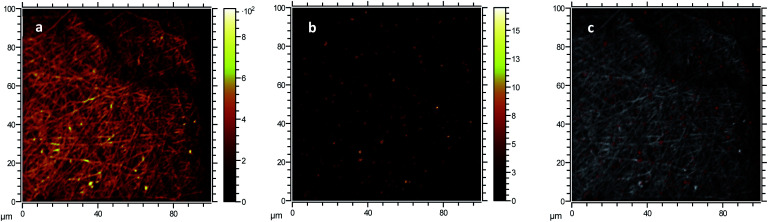
Morphological characterisation of PVA and CNP–PVA membranes. ToF-SIMS chemical images obtained from (a) the combination of secondary ions associated to PVA, (b) CeO^+^ ion associated to CNPs, and (c) an overlay of CNPs (red) and PVA (white) signals.

The formation of large agglomerates is a common feature of nanoparticle–polymer composites originated from *ex situ* incorporation of the nanomaterial^74^ and was attributed here to the unoptimised dispersion of CNPs into the PVA solution prior to electrospinning deposition. The presence of CNPs into the membranes was confirmed by ToF-SIMS analysis, whose mass spectra revealed a number of CeO_2_-related peaks, with CeO^+^ ion (*m*/*z* = 155.89) appearing to be the most abundant and common ionization product of CNPs in PVA. Please see Section S5† for the attribution of peaks in the ToF-SIMS spectra. Chemical images of PVA (Fig. 4a), CNPs (Fig. 4b), and their overlay (Fig. 4c) seem to confirm the findings of electron microscopy measurements, namely the PVA nanofibrous structure and the presence of large CNPs agglomerates, which are quite homogeneously distributed across the PVA membrane. Microscopy and surface analyses were non-conclusive regarding the full incorporation of CNPs into PVA nanofibres. On the one hand, TEM images showing small CNPs agglomerates suggested an effective coating (Fig. 3c), as the PVA layer surrounding the agglomerate was clearly observable. On the other hand, a polymer layer could not be observed for larger agglomerates (Fig. S4†). ToF-SIMS analysis could also provide some qualitative information on the coating of agglomerates. Being the analysis depth of ToF-SIMS < 2 nm, CeO_2_ could only be detected as in Fig. 4b if CNPs agglomerates are either non-coated or coated by a very thin PVA layer.

##### Composition and stability

**Fig. 5:**
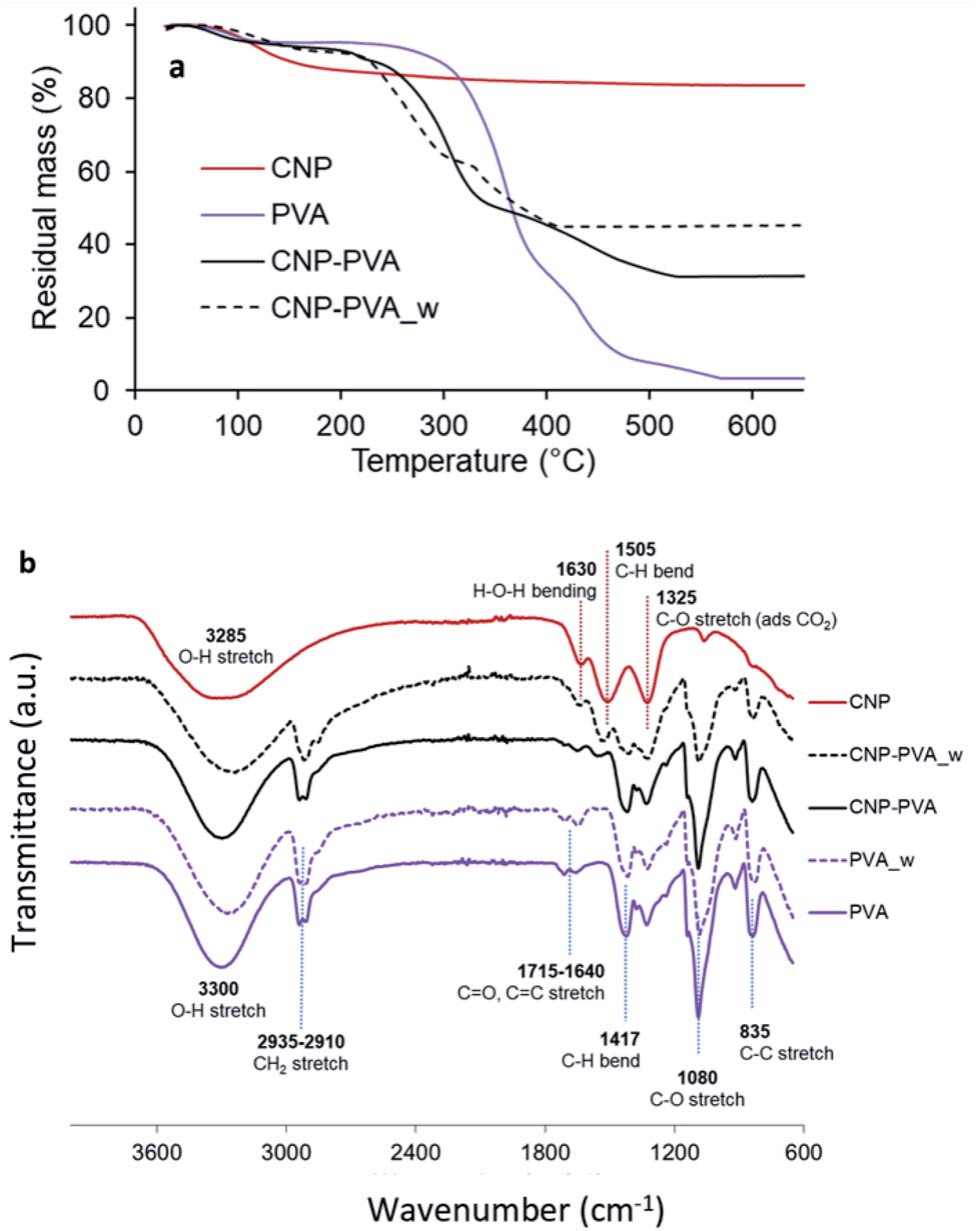
(a) TGA thermograms and (b) FTIR spectra of CNPs, pure PVA nanofibers and CNP–PVA (as such and after 4 h of immersion in DI water).

Fig. 5a shows the thermal stability of PVA, CNPs, and CNP–PVA investigated by TGA. The weight loss of CNPs up to 200 °C (11.2%) and between 200 and 500 °C (5.4%) was attributed to the loss of surface and structural water, respectively. The weight loss of pure PVA was ascribed to loss of moisture (at 98 °C), and to the gradual degradation of the polymer matrix over three steps after 250 °C.^75^ As expected for polymer nanocomposites, the thermal stability of CNP–PVA is considerably different than that of pure PVA. CNP–PVA membranes started to degrade at a lower temperature than pure PVA and exhibited a residual mass at 600 °C of 33%, which can be attributed to fully dehydrated CNPs.

The content of CNPs in the as spun CNP–PVA nanofibers can be estimated from the residual mass and resulted to be 37 ± 3 wt%. This result is in a good agreement with CNP content obtained by UV-assisted H_2_O_2_ digestion of CNP–PVA membranes, which was 33.6 ± 6.8 wt%. Unexpectedly, the thermal behaviour of water-exposed CNP–PVA membranes (CNP–PVA_w) was considerably different from the one of pristine CNP–PVA (Fig. 5a). More importantly, the residual mass of the water-exposed membrane was larger (46% compared to 33%). This result is compatible with an increased fraction of CNPs into the membrane, due to the loss of PVA mass during immersion in water (≈18%). This mass loss can be attributed to the solubilisation of PAA, Triton x-100 and to the release of low molecular weight PVA fractions. The increase in nanoceria content for water-exposed CNP–PVA was also qualitatively proved by FT-IR analysis (Fig. 5b). The FTIR spectrum of pristine CNP–PVA shows the features of both PVA and CNPs spectra. The bands at 3300 cm^−1^ 2930 cm^−1^ and the pattern in the range 1300–600 cm^−1^ are attributed to the PVA matrix,^76^ while the less intense band at 1500 cm^−1^ can be ascribed to contaminants adsorbed on CeO_2_ surface. Ethanol, carbon monoxide and other adventitious carbon species could have been adsorbed during preparation or storage. The characteristic CeO_2_ band attributed to Ce–O stretching is located at about 550 cm^−1^ (not visible in Fig. 5a). The FTIR spectrum of CNP–PVA_w shows a decrease of the intensity of the features attributed to PVA and the increase of the band at 1500 cm^−1^, thereby supporting an increase of the nanoceria content in the nanocomposite. The lack of a significant shifting of peaks suggests the absence of any relevant chemical interaction between nanoceria and the polymer. SEM analysis revealed that CNP–PVA undergoes a morphology change when immersed in water (Section S6†). This change, however, does not impair the use of CNP–PVA as adsorbents for REEs in repeated adsorption tests (paragraph 3.2.2).

### 3.2. Adsorption tests

#### 3.2.1. Static adsorption tests

##### Choice of pH for adsorption tests

**Fig. 6:**
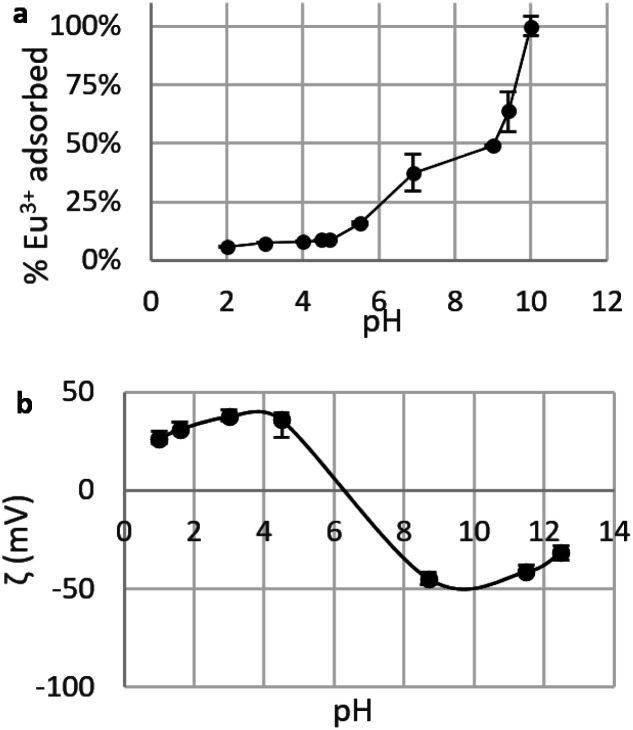
(a) Fraction of Eu^3+^ adsorbed by suspensions of CNPs at various pH (*c*_CeO_2__ = 0.25 g L^−1^, *c*_Eu^3+^,*t*=0_ = 8.4 mg L^−1^); (b) zeta potential *ζ* of CNPs at various pH (*c*_CeO_2__ = 0.5 g L^−1^, *c*_NaCl_ = 10 mM).

The choice of pH is considered to be the most important parameter when testing adsorption of metal ions by nano-adsorbents.^22^ To find the right experimental conditions , the influence of pH on adsorption was investigated by determining the Eu^3+^ fraction removed by CNPs in the pH range 2–10 (Fig. 6a). A pH < 2 was not used, as it leads to mobilisation of soluble Ce species, which would drastically change the adsorptive properties of the material.

Fig. 6a shows a clear increase of the removed fraction from pH 2 to 10, with Eu^3+^ completely adsorbed at pH 10. A similar trend was observed for UO_2_^2+^ adsorption by nanoceria^47^ and is reportedly caused by the nature of the interaction between REE ions and adsorbents, which is suggested to be purely electrostatic.^32,40,77^ The trend in Fig. 6 can be therefore explained in terms of occupied adsorption sites. In acidic environment (pH < 5), zeta potential measurements (Fig. 6b) showed that the CeO_2_ surface is positively charged (*ζ* = +20 to 40 mV): as protons are strongly adsorbed on the surface, the majority of binding sites are occupied and the fraction of adsorbed Eu^3+^ is very small (<10%). Near the point of zero charge pH_PZC_ (between 6 and 8) adsorbed Eu^3+^ fraction sharply increases while the adsorbent's surface becomes less positively charged and the competition for binding sites is smaller. At alkaline pH, the surface is negatively charged and Eu^3+^ adsorption is theoretically more favourable due to electrostatic attraction. However, at pH > 6 hydrolysis takes place^77,78^ and hydroxy species such as Eu(OH)^2+^, Eu(OH)_3_, and Eu(OH)_4_^−^ are dominant.^79^ Therefore, the high adsorption values at pH > 6 are expected to reflect a change in Eu^3+^ speciation that can lead to adsorption-independent removal of Eu^3+^ from the solution. For this reason, in this work the adsorption tests are conducted at near-neutral pH (5.8).

##### Adsorption isotherms

**Fig. 7:**
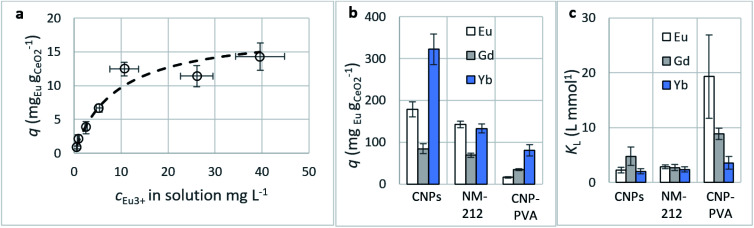
(a) Adsorption of Eu^3+^ from CNP–PVA: equilibrium adsorption data (empty dots) and adsorption isotherm determined *via* the Langmuir model (dashed line). (b) Maximum adsorption capacity *q*_M_ and (c) Langmuir equilibrium constant *K*_L_ obtained *via* fitting of adsorption data with the Langmuir equation. *K*_L_ units are here L mmol^−1^ to facilitate comparison between the various REEs. *c*_CeO_2__ = 0.25 g L^−1^, *c*_REE,*t*=0_ = 2–150 mg L^−1^.

A summary of the Freundlich and Langmuir parameters derived from the fitting of adsorption data (as for Fig. 7a) is given in Table 2.

**Table 2:** Langmuir and Freundlich parameters from Eu^3+^, Gd^3+^, and Yb^3+^ adsorption tests. *c*_CeO_2__ = 0.25 g L^−1^, *c*_REE,t=0_ = 0.5–150 mg L^−1^

	Eu^3+^ adsorption			Gd^3+^ adsorption			Yb^3+^ adsorption		
	CNPs	NM-212	CNP–PVA	CNPs	NM-212	CNP–PVA	CNPs	NM-212	CNP–PVA
**Langmuir**									
*q*_M_ (mg_REE_ g_CeO_2__^−1^)	178.2 ± 18.1	142.8 ± 8.2	16.1 ± 1.6	84.6 ± 12	69.1 ± 4.9	34.7 ± 2.1	322.2 ± 36.5	132.5 ± 10.7	81.1 ± 13.7
*K*_L_ (L g_REE_^−1^)	15 ± 3.4	18.7 ± 2.2	127 ± 50.2	30.2 ± 10.7	17.3 ± 3.4	56.3 ± 6.7	11.7 ± 2.8	13.7 ± 2.9	20.6 ± 6.7
*R* ^2^	0.98	0.99	0.95	0.94	0.98	0.99	0.98	0.98	0.98

**Freundlich**									
*K*_F_ (mg_REE_^(1−1/*n*)^ g_CeO_2__^−1^ L^1/*n*^)	8.3 ± 1.6	5.7 ± 1.3	1.9 ± 0.2	4.1 ± 1.6	3.8 ± 0.6	2.7 ± 0.3	5.6 ± 2.8	3.9 ± 1.7	3 ± 1.1
*n* (dimensionless)	1.8 ± 0.1	1.6 ± 0.1	1.3 ± 0.1	1.5 ± 0.2	2.1 ± 0.1	1.6 ± 0.1	1.3 ± 0.2	1.6 ± 0.2	1.5 ± 0.2
*R* ^2^	0.98	0.98	0.98	0.91	0.99	0.99	0.98	0.95	0.96

###### Adsorption capacity

Fig. 7b summarises the values of the maximum adsorption capacity *q*_M_ from Table 2. *q*_M_ ranged between 16 and 322 mg_REE_ g_CeO2_^−1^, with the highest value reported for the Yb^3+^ adsorption by CNPs (≈322 mg_Yb_ g_CeO2_^−1^). Taking into consideration the adsorption of the same REE from various adsorptive materials, values of *q*_M_ followed the trend CNPs > NM-212 > CNP–PVA. A similar trend can be observed also with the Freundlich equilibrium constant *K*_F_ (Section S7†). Even if the Freundlich equation does not predict an adsorption maximum, *K*_F_ represents the adsorbed concentration at the equilibrium when the concentration of the adsorbate in solution is unitary (= 1 mg L^−1^) and can therefore be used to compare adsorption isotherms obtained in similar conditions. The higher *q*_M_ values of CNPs compared to NM-212 can be reasonably ascribed to its smaller particle size and the subsequent larger surface area available for adsorption. The smallest *q*_M_ values for CNP–PVA are likely caused by a combination of CNPs agglomeration and polymer–CeO_2_ interaction, which is known to decrease the surface area of nanomaterials in nanocomposites.^80^ Interestingly, the *q*_M_ values found in this work are similar to those reported in recent adsorption studies conducted with nanoceria in similar experimental conditions. For example, the maximum adsorption capacity of UO_2_^2+^ by CeO_2_ nanoparticles was reported to be ≈0.98 and ≈1.62 mmol g_CeO2_^−1^ ,^47,48^ whereas the *q*_M_ found in this work ranges from 0.5 to 1.8 mmol_REE_ g_CeO2_^−1^. This could suggest that the uptake mechanism of lanthanide and actinide ions from CeO_2_ is similar. The higher *q*_M_ values found for Yb^3+^ could hint to an increased adsorption capacity of heavy REEs compared to mid and light REE ions. The interaction between REE trivalent ions and oxygen-containing hard bases is believed to be largely ionic^77^ and the decrease in ionic radii across the lanthanide series should results in an increase of the ionic interaction with the adsorbent.^81^

###### Affinity of adsorbate

The Langmuir equilibrium constant *K*_L_ ranges between 11.7 and 127.0 L g^−1^ (Table 2). *R*_L_ values calculated according to eqn (6) are, therefore, in the range 0.10–0.98 and show that the adsorption of REEs on the nanoceria-based materials used in this work is a favourable process. However, there seem to be considerable differences between *K*_L_ determined for the various adsorbents. If *K*_L_ values are expressed in terms of L mmol^−1^ to ensure comparison between adsorbents (Fig. 7c), *K*_L_ values for CNP–PVA (up to 19.5 L mmol^−1^ for Eu^3+^) are considerably larger than for CNPs and NM-212 (2.0 to 4.5 L mmol^−1^). As *K*_L_ can be understood as a measure of the adsorbate–adsorbent affinity,^82^ results suggest a more favoured adsorption process when nanoceria is embedded in the PVA fibres. A potential reason could be a synergistic effect of the PVA matrix due to the stabilising effect of –OH pendants located in the vicinity of adsorption sites.

#### 3.2.2. Repeated use of adsorbents

Even if static adsorption experiments are a useful tool to assess the potential of adsorbents, they do not necessarily reflect the behaviour of adsorbents under the conditions of the intended final application. For example, in case of large volumes of REE-containing water streams with competing ions for adsorption sites (*e.g.*, Cu^2+^ from processed WEEE), continuous-flow or multi-step batch adsorption systems could be preferred over static systems, due to their easier preparation and the possibility to add sample pre-treatment steps for the removal of competing ions. To investigate the behaviour of the adsorbent under cycling and continuous-flow use, repeated static and dynamic adsorption experiments were carried out.

##### Static arrangement

**Fig. 8:**
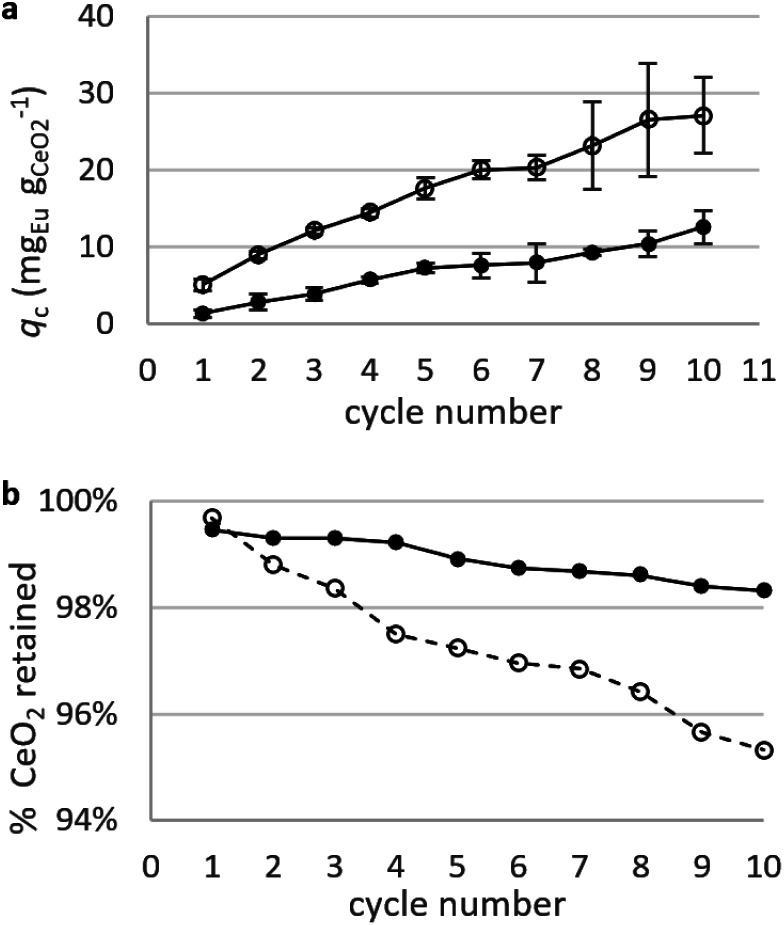
Adsorption Eu^3+^ in repeated static adsorption tests. (a) Adsorption capacity of Eu^3+^ with CNPs (empty dots) and CNP–PVA (filled dots). (b) Percentage of nanoceria retained after each adsorption cycle. Initial *m*_CeO_2__ = 1.3 mg (CNP) and 2.5 mg (CNP–PVA); *c*_Eu_ for each step = 5 mg L^−1^.

CNPs and CNP–PVA were used in repeated adsorption cycles of Eu^3+^, and the equilibrium adsorption capacity *q*_e_ monitored throughout the experiment. Fig. 8a shows the cumulative adsorption capacity for each step. Both materials were effective in adsorbing the target REE throughout the experiment, reaching a final *q*_c_ ≈ 27 mg_Eu_ g_CeO2_^−1^ (CNPs) and 12 mg_Eu_ g_CeO2_^−1^ (CNP–PVA) after 10 adsorption cycles. These values are smaller than *q*_M_ derived from the isotherms, with CNPs showing the larger decrease (6 times lower, from *q*_M_ ≈ 180 mg_Eu_ g_CeO2_^−1^ to *q*_c,final_ ≈ mg_Eu^3+^_ g_CeO2_^−1^) compared to CNP–PVA (from *q*_M_ ≈ 16 mg_Eu_ g_CeO2_^−1^ to *q*_c,final_ ≈ 12 mg_Eu_ g_CeO2_^−1^). This finding could be attributed to the different experimental conditions. Under repeated use, the occurrence of competing adsorption and desorption processes, the change in the agglomeration state of nanoparticles and loss of CeO_2_ between cycles could influence the adsorption capacity of adsorbents. The loss of CeO_2_ was monitored during the experiment, by detecting cerium in the solution after separation from the adsorbent (Fig. 8b). After the last cycle, ≈95% and ≈98% of nanoceria's weight was retained by CNPs and CNP–PVA, respectively. The larger loss observed for CNPs was ascribed to the permeation of small, non-agglomerated nanoparticles through the ultra-centrifugal filters used during the separation step at the end of each cycle. This effect is avoided by using CNP–PVA, as nanoparticles are effectively incorporated into the polymer matrix. Therefore, CNP–PVA seems a more promising adsorbent for a repeated or continuous use. CNP–PVA membranes were used to test the adsorbent regeneration *via* desorption of REEs. Desorption is usually achieved by adding a mineral acid, thereby prompting the replacement of adsorbed REE ions with protons.^32^ Therefore, repeated desorption cycles were carried out by dipping CNP–PVA membranes in deionized water at pH 2 (HNO_3_ 0.01 M). Although the recovery of Eu^3+^ was already ≈25% after the first desorption step, following steps could only recover roughly 1% Eu^3+^ each to a total of ≈30% after 5 cycles.

Regeneration of CNPs resulted in 50% of adsorbate already released after the first step. These results suggest the need for stronger desorption process to regenerate CNP–PVA. An improved release of REE ions could be achieved by flushing the membrane with an acidic solution over a long time (promoting a “dynamic” desorption) or by using REE-complexing molecules (*e.g.* TBP or EDTA).^83,84^

##### Dynamic arrangement

**Fig. 9:**
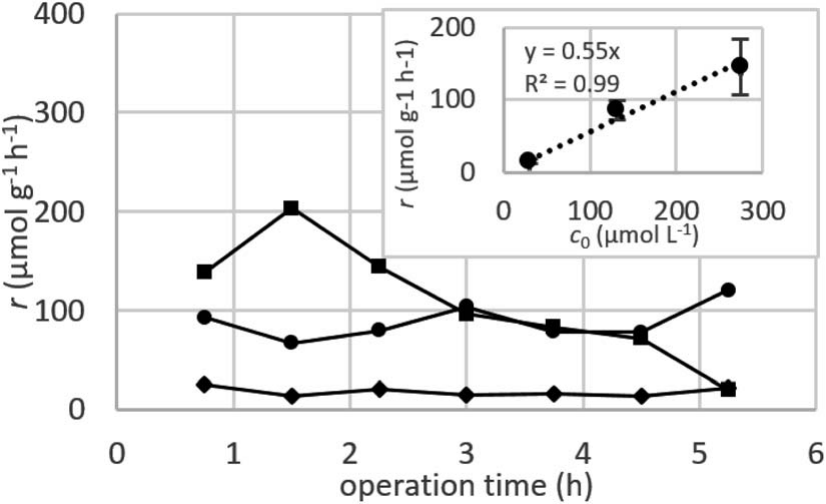
Adsorption rate determined in a continuous-flow system with various initial Eu^3+^ concentrations *c*_0_: 3 mg L^−1^ (diamond dots), 20 mg L^−1^ (round dots) and 40 mg L^−1^ (square dots). Chart at the upper right corner: plot of initial *r versus c*_0_. *m*_CeO_2__ = 1 to 3 mg, *F* = 0.1 mL min^−1^.

While static experiments are generally useful to assess the basic potential of an adsorbent, dynamic experiments are often closer to their intended final use. Dynamic studies are a useful way to adjust the method conditions in the light of full-scale adsorption process, because it can shed light on the adsorption mechanism and potential rate-controlling steps such as mass transport effects.^85^ In this work, CNP–PVA membranes were tested in continuous mode over few hours, by feeding in a solution with a fixed Eu^3+^ concentration (3, 20 or 40 mg L^−1^). Fig. 9 shows the adsorption rate determined with eqn (9) for various Eu^3+^ concentrations in the feed. As expected, the initial rate increases with the concentration of Eu^3+^ in the feed. After only 2 hours of operation time, however, the adsorption rate determined for the highest Eu^3+^ concentration sharply decreases, suggesting that the maximum uptake capacity of the system was already reached. The plot of the initial adsorption rate against the Eu^3+^ concentration in the feed (small box in Fig. 9) gives a straight line, indicating a first-order process,^86^ with the observed adsorption coefficient *k* equal to the slope of the regression line (*k* = 0.55 L g^−1^ h^−1^). It is worth noting that the observed first-order process can be ascribed not only to adsorption kinetics, but also to mass-transport effects. In the presented system, the process of adsorption entails the diffusion from the bulk to PVA surface and from PVA surface to CeO_2_ surface. Any of these steps could be controlling the overall adsorption kinetics. The observed adsorption coefficient was used to determine the required “size” of the system,^69^ that is the amount of adsorbent needed to adsorb a given Eu^3+^ fraction, *via*eqn (10).10
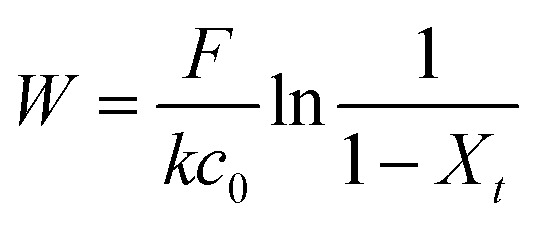


This means that, in order to adsorb 90% of europium from a 3 mg L^−1^ solution, ≈166 mg of CNPs (≈420 mg of CNP–PVA) are needed, much higher that what was used in the experiment (≈2 mg). Therefore, the use of a larger quantity of CNP–PVA, for example in sequential filtering modules, is needed to achieve the adsorption of all Eu^3+^. Alternatively, a recycle system with adsorption–desorption cycles could be used.

## 4. Conclusions

This work investigated the potential of water-suspended and polymer-embedded cerium oxide nanoparticles in the adsorption of REEs from aqueous solution. Ultra-small cerium oxide nanoparticles (CNPs, *d* ≈ 3 nm) were produced and effectively embedded into PVA *via* electrospinning. Resulting CNP–PVA nanocomposites were formed by woven–non-woven PVA nanofibres (*d* = 280 nm) housing CNPs agglomerates (size from 50 to 2400 nm) with a nanoceria content in PVA of ≈34 wt%. Surface analysis showed that CNPs agglomerates were homogeneously distributed across the PVA membrane but was non-conclusive regarding their entire incorporation into the nanofibres. However, it was proved that the most of nanoceria is retained by the membrane during adsorption tests. CNPs, CNP–PVA and the benchmark material NM-212 effectively absorbed REEs from model water solutions. The maximum adsorption capacities *q*_M_ were determined by fitting the single-element adsorption isotherms with the Langmuir equation and ranged between 16 and 322 mg_REE_ g_CeO2_^−1^, with a *q*_M_ trend CNPs > NM-212 > CNP–PVA. The largest value was found for the adsorption of Yb^3+^ by CNPs (≈322 mg_Yb_ g_CeO2_^−1^). CNP–PVA could successfully adsorb Eu^3+^ in repeated adsorption cycles, showing a minimal loss of adsorptive material that was lower than water-suspended CNPs. CNP–PVA could remove Eu^3+^ under a continuous flow configuration, where adsorption followed a first-order process with an observed kinetic coefficient *k* = 0.55 L g^−1^ h^−1^. Regeneration of CNP–PVA adsorbent *via* mild acidic treatment proved to be only partially effective, with only 30% of the REE recovered.

For the first time, this work explores the use of the nanoceria–polymer nanocomposite CNP–PVA as adsorbent for REE ions. Even if water suspended CNPs proved to be more effective adsorbents, their use in practice would be hindered by the challenging separation process required to recover the adsorbent from the water phase. Compared to uncoated nanoceria, CNP–PVA membranes have the crucial advantage to confine the adsorptive material within the polymer nanofibres, thereby preventing any major loss during use and facilitating continuous-flow applications, regeneration, and reuse. At the same time, the swelling of PVA in water ensures that adsorption sites on nanoceria are easily accessible by REE ions. Based on the results of this study, CNP–PVA membranes could find application as a downstream separation process in the recovery of strategically important REEs from electronic waste. Moreover, owing to the physicochemical similarities between lanthanides and actinides, such adsorbents could be used to remove radioactive actinide elements from ground or surface waters. To fully exploit the potential of CNP–PVA as adsorbent, further research efforts should focus on developing a suitable desorption strategy and on optimising their preparation procedure, aimed at improving the filler's dispersion in the polymer and the structural integrity of the nanofibres during use.

## Conflicts of interest

There are no conflicts to declare.

## Supplementary Material

RA-011-D1RA02097H-s001
