# Supplementary material for: Recovery of rare earth elements by nanometric CeO2 embedded into electrospun PVA nanofibres
Source: RSC Adv. 2021 May 28;11(32):19351–62. doi: 10.1039/d1ra02097h (PMC9033589; doi:10.1039/d1ra02097h)
Supplement: RA-011-D1RA02097H-s001 [file RA-011-D1RA02097H-s001.pdf]

Electronic Supplementary Material (ESI) for RSC Advances.
This journal is © The Royal Society of Chemistry 2021

Supplementary information

Recovery of rare earth elements by nanometric CeO_2 embedded into electrospun PVA nanofibres

S1. Optical size of CNP

Determination of the band gap. The band gap of semiconductors that show negligible absorbance in the sub-band gap energy range can be estimated via Equation S1

$$(\alpha E_{phot})^n = C(E_{phot} - E_g) \qquad \text{Eq. (S1)}$$

Where α is the absorption coefficient, E_{phot} is the photon energy (=*hv*), *C* is a material-dependent constant and *n* is 2 for direct transition and ½ for indirect transitions.. The absorption coefficient α can be determined from UV-Vis spectral data (Taguchi et al. 2009, Lin and Chowdhury 2010) as

$$\alpha = \frac{2.303\ x\ 10^3 A\rho}{lc} \qquad \text{Eq. (S2)}$$

Where *A* is the absorbance, *ρ* is CeO_2 (= 7.28 g cm^{-3}), *l* is the optical path length (= 1 cm) and *c* is the concentration of ceria suspension (g mL^{-1}). The intersection of the extrapolated linear part of of Equation S1 (Tauc plot) gives an estimation of the optical band gap (Figure S1b).

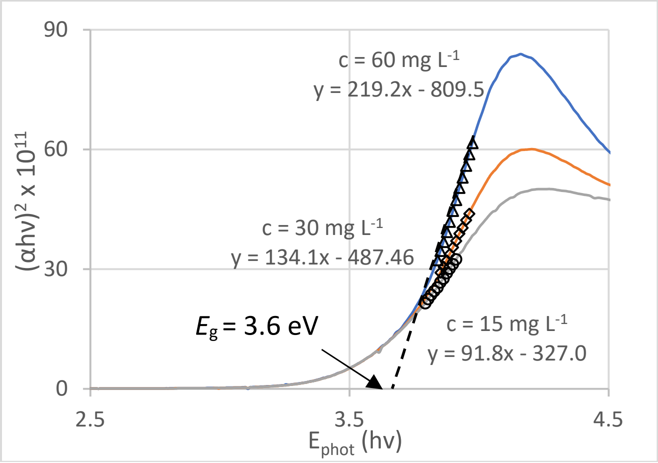

Figure S1. UV-Vis spectra of CNP (a) and the corresponding Tauc plots (b) at 15, 30, and 60 mg L^{-1} (grey, orange and blue curves, respectively)

Determination of optical particle size. When the particle size becomes smaller than the exciton radius, i.e. ≈ 8 nm for CeO_2 (Ramasamy, Mohana and Rajendran 2018), quantum confinement leads to size-dependent enlargement of the band gap and results in a blue-shift in the absorption edge. The particle size can be determined by the following equation (Goharshadi, Samiee and Nancarrow 2011)

$$E_g = E_{g,b} + \frac{\pi\hbar}{2r^2}\left(\frac{1}{m_e} + \frac{1}{m_h}\right) - \frac{1.8e^2}{\delta r} \qquad \text{Eq. (S3)}$$

Where $E_{g,b}$ is the bulk band gap (3.19 eV for ceria), *r* is the particle radius (m), $\hbar$ is the reduced plank constant, δ is the bulk dielectric constant and m_e and m_h are the effective masses of electron and hole, respectively. They can both be expressed based on the mass of free electron m_0, as $m_e = m_h = 0.4m_0$. When *r* is very small (comparable to Bohr radius), the third factor in the equation can be neglected. As all variables of Eq. S3 are known, the equation can be solved for *r*, yeilding *r* = 2.3 nm. This value is considered to be the median of a number-average particle size (Dieckmann et al. 2009).

S2. Sketch of dynamic adsorpion experiments

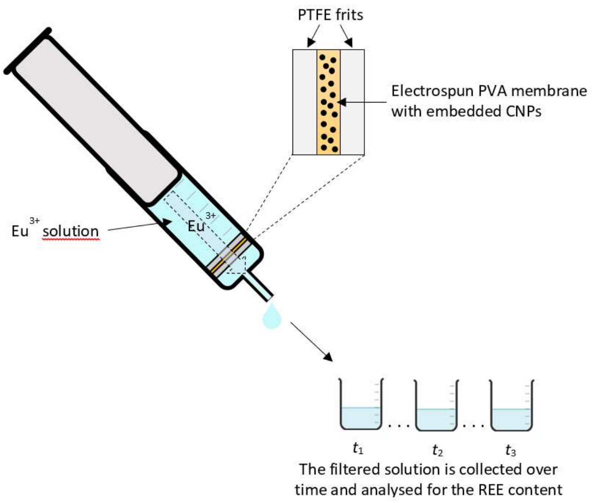

Figure S2. Sketch of the system used to test CNP-PVA in dynamic adsorption experiments.

S3. Physico-chemical characterisation of NM-212

The material NM-212 has been the subject of extensive characterisation efforts (Singh et al. 2014, Gosens et al. 2014, Römer et al. 2019). A summary of NM-212 physico-chemical properties is given in Table S1.

Table S1. Physico-chemical characterisation of NM-212

physio-chemical property	Technique	Measured property	Value
Particle size	SEM[1]	d_{TEM} median value of the particle size distribution	28.4 ± 10.4 nm
Particle shape	TEM[1]	-	polyhedral particles with variable sizes and morphology
Agglomeration size in suspension	AF-FFF/DLS[2]	d_H z-average hydrodynamic diameter	174.9 ± 14.4 nm
SSA	N_2 adsorption[2]	S_{BET}	28.3 ± 0.3 m^2 g^{-1}

[1](Singh et al. 2014), [2]measured in this study

Figure S3 shows the elugram determined with AF-FFF by UV-Vis detection at λ = 300 nm and the calculated *z*-average hydrodynamic diameter d_H determined via DLS. The measured d_H ranged between 100 and 350 nm, with an average d_H = 174 nm and a PDI = 0.02. The average d_H was calculated by averaging the d_H values determined by the full width at half maximum FWHM of the UV-Vis elugram (Caputo et al. 2019). The PDI was calculated as $(\sigma/d_{H,mean})^2$ where $d_{H,mean}$ is the mean d_H value and σ is its standard error.

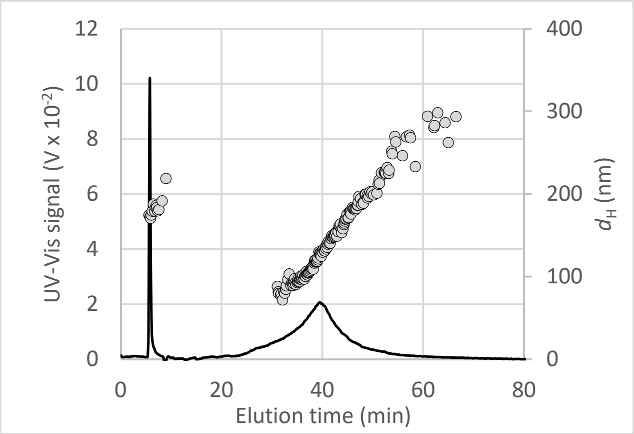

Figure S3. Outcome of AF-FFF measurements of NM-212, with the elugram detected UV-Vis detector (continuous line) and d_H calculated by DLS (dots).

S4. Evaluation of TEM micrographs

CNPs. Particle identification was carried out with the ImageJ software using the Particle Sizer plugin and setting ellipsoid as fitting shape. Selected statistical parameters of the size distribution (mean, median, PDI) are shown in the table below. The particles' specific surface area was found by applying Eq. S4 (Baldim et al. 2018)

$$SSA = \frac{6\exp\left(-2.5s^2\right)}{D\rho} \qquad \text{Eq. (S4)}$$

Where s is the dispersity of the distribution, *D* is the median Feret min diameter and ρ is the CeO_2 density (≈ 7.28 g cm^{-3}).

CNP-PVA. Unlike for CNP, the short and long axis length of nanoceria agglomerates in PVA fibres were determined manually. Figure S4b reports a selection of TEM micrographs showing nanoceria agglomerates.

median Feret min diameter	2.97 nm
standard deviation	1.02 nm
mean Feret min diameter	3.16 nm
dispersity	0.32
SSA as from Eq. (S4)	214.1 $m^2 g^{-1}$

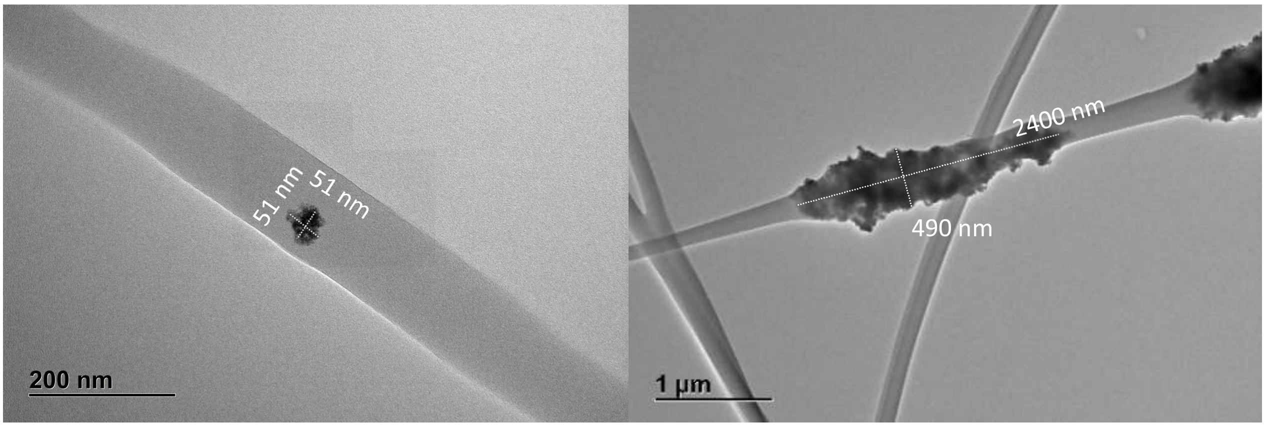

Figure S4b. TEM micrographs of CNP-PVA nanofibres with nanoceria agglomerates.

S5 ToF-SIMS mass spectrum of CNP-PVA

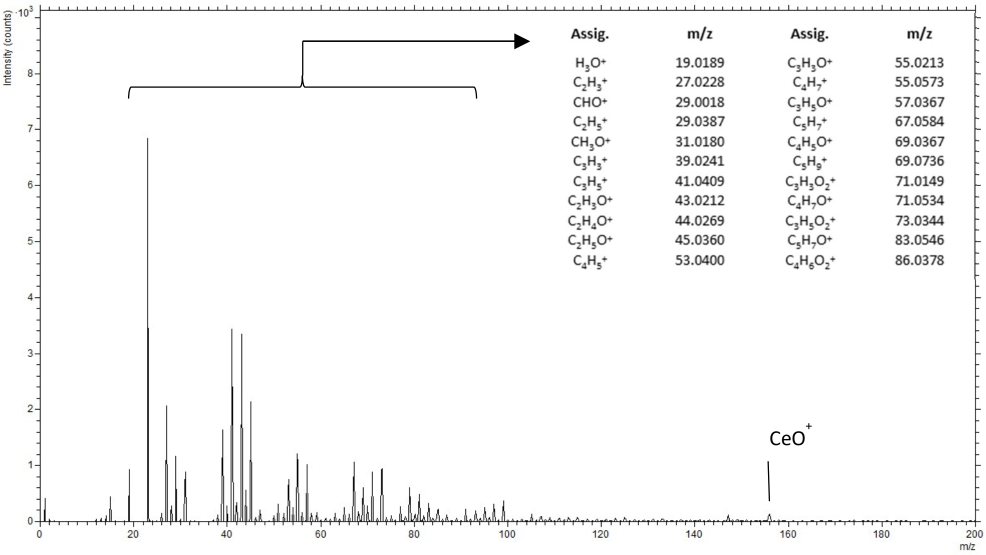

Assig.	m/z	Assig.	m/z
H_3O^+	19.0189	$C_3H_3O^+$	55.0213
$C_2H_3^+$	27.0228	$C_4H_7^+$	55.0573
CHO^+	29.0018	$C_3H_5O^+$	57.0367
$C_2H_5^+$	29.0387	$C_5H_7^+$	67.0584
CH_3O^+	31.0180	$C_4H_5O^+$	69.0367
$C_3H_3^+$	39.0241	$C_5H_9^+$	69.0736
$C_3H_5^+$	41.0409	$C_3H_3O_2^+$	71.0149
$C_2H_3O^+$	43.0212	$C_4H_7O^+$	71.0534
$C_2H_4O^+$	44.0269	$C_3H_5O_2^+$	73.0344
$C_2H_5O^+$	45.0360	$C_5H_7O^+$	83.0546
$C_4H_5^+$	53.0400	$C_4H_6O_2^+$	86.0378

Figure S5. ToF-SIMS mass spectrum of CNP-PVA with peaks associated to PVA (Endo et al. 1997) and CNPs (CeO^+).

S6. Morphological change of CNP_PVA upon water immersion

SEM micrographs of as spun CNP-PVA and CNP-PVA_w (after 4-hour immersion in water) are reported in Figure S6a. In water, PVA electrospun membranes underwent a morphological reorganization from solid fibers to a highly hydrated gel, which collapsed once they were left drying. However, CNP agglomerates remained enclosed in the newly formed PVA hydrogel.

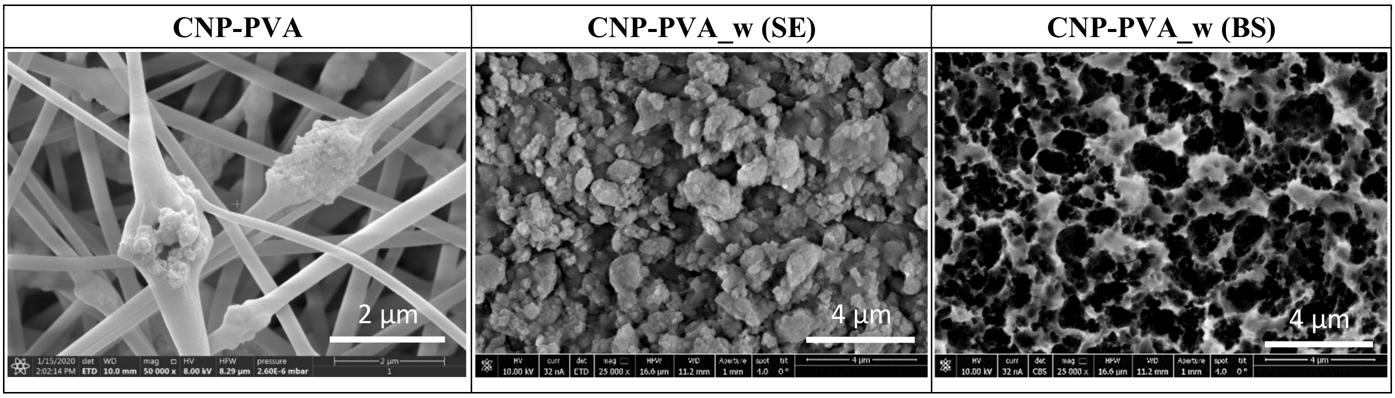

Figure S6a. SEM micrographs of CNP-PVA: as spun CNP-PVA nanofibers (left), and after water immersion imaged using secondary electrons (center) and backscattered electrons (right) with inverted colours (PVA: white)

PVA electrospun membranes have a very high specific surface area and swell extensively in water. Due to the loss of soluble components, the mass of the membrane decreases (Figure S6b). After 4 hours of water exposure, pure PVA membranes have lost 35% of the original mass, while CNP-PVA membranes have lost only 18%. This finding suggests that the mass loss can be mostly ascribed to PVA and not to CNPs.

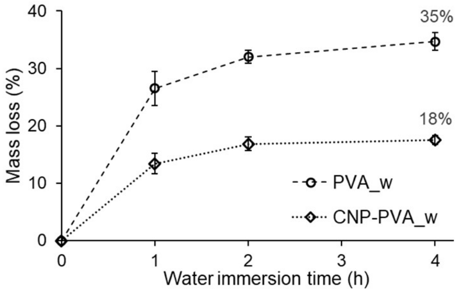

Figure S6b. Membrane mass loss due to solubilisation and dissolution in water

S7. Freundlich equilibrium constants

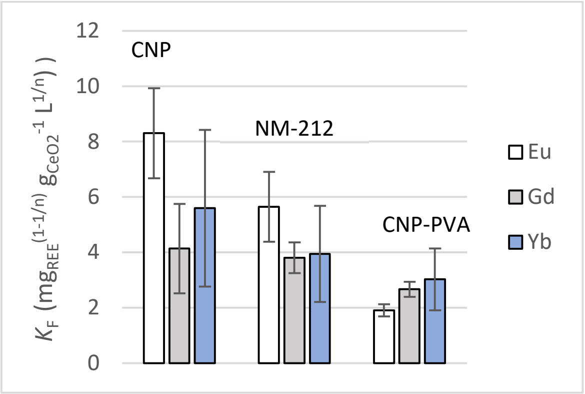

Figure S7. Freundlich parameter K_F obtained via fitting of adsorption data with the Freundlich equation.

Reference

Baldim, V., F. Bedioui, N. Mignet, I. Margaill & J.-F. Berret (2018) The enzyme-like catalytic activity of cerium oxide nanoparticles and its dependency on Ce 3+ surface area concentration. *Nanoscale,* 10**,** 6971-6980.

Caputo, F., A. Arnould, M. Bacia, W. L. Ling, E. Rustique, I. Texier, A. P. Mello & A.-C. Couffin (2019) Measuring particle size distribution by asymmetric flow field flow fractionation: a powerful method for the preclinical characterization of lipid-based nanoparticles. *Molecular pharmaceutics,* 16**,** 756-767.

Dieckmann, Y., H. Cölfen, H. Hofmann & A. Petri-Fink (2009) Particle size distribution measurements of manganese-doped ZnS nanoparticles. *Analytical chemistry,* 81**,** 3889-3895.

Endo, K., T. Hoshi, N. Kobayashi, H. Miura & M. Kudo (1997) Spectral analysis of eight polymers in SIMS by MO calculation. Prediction of cleavage of polymers and structural formula of the positive-ion fragment. *Polymer journal,* 29**,** 457-466.

Goharshadi, E. K., S. Samiee & P. Nancarrow (2011) Fabrication of cerium oxide nanoparticles: characterization and optical properties. *Journal of colloid and interface science,* 356**,** 473-480.

Gosens, I., L. E. Mathijssen, B. G. Bokkers, H. Muijser & F. R. Cassee (2014) Comparative hazard identification of nano- and micro-sized cerium oxide particles based on 28-day inhalation studies in rats. *Nanotoxicology,* 8**,** 643-653.

Lin, K.-S. & S. Chowdhury (2010) Synthesis, characterization, and application of 1-D cerium oxide nanomaterials: a review. *International journal of molecular sciences,* 11**,** 3226-3251.

Ramasamy, V., V. Mohana & V. Rajendran (2018) Characterization of Ca doped CeO2 quantum dots and their applications in photocatalytic degradation. *OpenNano,* 3**,** 38-47.

Römer, I., S. M. Briffa, Y. Arroyo Rojas Dasilva, D. Hapiuk, V. Trouillet, R. E. Palmer & E. Valsami-Jones (2019) Impact of particle size, oxidation state and capping agent of different cerium dioxide nanoparticles on the phosphate-induced transformations at different pH and concentration. *PloS one,* 14**,** e0217483.

Singh, C., S. Friedrichs, G. Ceccone, N. Gibson, K. A. Jensen, M. Levin, H. Goenaga Infante, D. Carlander & K. Rasmussen (2014) Cerium Dioxide, NM-211, NM-212, NM-213. Characterisation and test item preparation. *JRC Repository: NM-Series of Representative Manufactured Nanomaterials*.

Taguchi, M., S. Takami, T. Naka & T. Adschiri (2009) Growth mechanism and surface chemical characteristics of dicarboxylic acid-modified CeO2 nanocrystals produced in supercritical water: tailor-made water-soluble CeO2 nanocrystals. *Crystal growth & design,* 9**,** 5297-5303.
